# Establishment of an *Ex Vivo* Tissue Culture Model for Evaluation of Antitumor Efficacy in Clear Cell Renal Cell Carcinoma

**DOI:** 10.3389/fonc.2022.851191

**Published:** 2022-04-06

**Authors:** Shanjuan Hong, Qing Yuan, Haizhui Xia, Yuan Dou, Tiantian Sun, Tian Xie, Zhiyin Zhang, Wei He, Chen Dong, Jian Lu, Li Guo, Ling Ni

**Affiliations:** ^1^ Institute for Immunology and School of Medicine, Tsinghua University, Beijing, China; ^2^ Department of Urology, The Third Medical Center of Chinese Peoples Liberation Army (PLA) General Hospital, Beijing, China; ^3^ Department of Urology, Peking University Third Hospital, Beijing, China; ^4^ R&D Center, Suzhou Kanova Biopharmaceutical Co., Ltd., Suzhou, China; ^5^ Center for Human Disease Immuno-Monitoring, Beijing Friendship Hospital, Beijing, China

**Keywords:** VISTA, ccRCC, PD-1, immunotherapy, TNF-α, *ex vivo*

## Abstract

There are many potential immunotherapeutic targets for cancer immunotherapy, which should be assessed for efficacy before they enter clinical trials. Here we established an *ex vivo* cultured patient-derived tumor tissue model to evaluate antitumor effectiveness of one VISTA inhibitor, given that our previous study showed that VISTA was selectively highly expressed in human clear cell renal cell carcinoma (ccRCC) tumors. We observed that all the tested patients responded to the anti-VISTA monoclonal antibody as manifested by TNF-α production, but only a small fraction were responders to the anti-PD-1 antibody. Co-blockade of VISTA and PD-1 resulted in a synergistic effect in 20% of RCC patients. Taken together, these findings indicate that this *ex vivo* tumor slice culture model represents a viable tool to evaluate antitumor efficacies for the inhibitors of immune checkpoints and further supports that VISTA could serve as a promising target for immunotherapy in ccRCC.

## Introduction

Renal cell carcinoma (RCC) is the most common type of kidney cancer, and clear cell RCC (ccRCC) is the main type of RCC (1). Currently, RCC accounts for approximately 4% of all adult malignancy with an increasing incidence. An estimated 403,262 new cases of RCC are diagnosed worldwide (2), and approximately 30% of the patients present with metastatic disease at the time of diagnosis (3).

Immunotherapy has been exploited to treat RCC patients. In 2015, the immune checkpoint inhibitor (anti-PD-1 antibody, nivolumab) was granted approval by the Food and Drug Administration (FDA) as the second-line treatment for advanced RCC based on the CheckMate-025 trial (4). However, the response rate to anti-PD-1 antibodies is approximately 25%. In 2018, the FDA approved the combination of anti-PD-1 and anti-CTLA-4 as a frontline treatment for intermediate- and poor-risk patients with advanced RCC based on phase III CheckMate-214 trial (5). Anti-CTLA-4 enhances T-cell priming against tumor antigens, while anti-PD-1 enhances the metabolism and effector function of tumor-specific progenitor exhausted T cells (6). The combination of those two antibodies induced a maximal antitumor effector phenotype and, however, was associated with significant toxicity in more than half of patients with grade 3 or 4 adverse events (5, 7). Therefore, there is an urgent need to find another target for RCC immunotherapy. Accordingly, novel models are required to assess the efficacy of the potential targets.

VISTA (also known as B7-H5, GI24, Dies1, and PD-1 homolog) is one of the checkpoint molecules (8, 9) with an extracellular domain homologous to PD-L1 (10). Human VISTA is highly expressed on myeloid cells, while CD4^+^ and CD8^+^ T cells express moderate levels. VISTA signaling exerts a suppressing effect on T-cell activation under physiological conditions (11). VISTA is expressed by only certain subtypes of tumor cells, such as gastric cancer cells (12), colorectal cancer cells (13), and oral squamous cell carcinoma (14). Of interest, the treatment of melanoma patients with anti-PD-1 led to VISTA upregulation, eliciting adaptive resistance of PD-1 blockade (15). During the treatment of patients with prostate cancer with anti-CTLA-4 antibodies, an increase in VISTA expression was also detected, accounting for the resistance of anti-CTLA-4 therapy (16). One report (17) showed that anti-PD-1 or anti-CTLA-4 alone resulted in a more than 2-fold increase in VISTA-expressing CD4^+^ T-cell infiltrate, whereas the combined therapy enhanced 4-fold in a T3 murine methylcholanthrene (MCA)-induced sarcoma model. These observations indicated that VISTA might be associated with adaptive resistance of immune checkpoint inhibitor therapy.

Previously, we found that VISTA mRNA and protein were selectively highly expressed in human ccRCC tumors, mostly on tumor-associated macrophages (TAMs) (18). In addition, VISTA expression was strongly correlated with poor CD8^+^ T-cell responses, and VISTA blockade resulted in significantly reduced growth of murine RENCA RCC model (18). Here we established one *ex vivo* tumor tissue culture model to assess antitumor efficacies of new candidate targets, such as anti-VISTA antibodies. The use of the *ex vivo* tissue culture system will facilitate screening of the antitumor immune response in individual patient-derived tumor tissues, especially immune system-targeted agents.

## Methods and Materials

### Patients and Specimens

Fresh tumor samples, para-tumors, and matched blood without any preoperative therapies were obtained from patients undergoing nephrectomy or renal partial resection in the 8th Medical Center of Chinese PLA General Hospital and Peking University Third Hospital. All the procedures in this study were approved by the institutional review board at Tsinghua University and were performed in line with the institutional guidelines.

### Isolation of Peripheral Blood Mononuclear Cells (PBMCs) and Tissue/Tumor-Infiltrating Leukocytes

Blood from ccRCC patients was drawn into heparinized tubes and centrifuged on Ficoll-Hypaque gradients (GE Healthcare Life Sciences, Chicago, IL, USA). Fresh tumors from ccRCC patients were digested with 1 mg/ml of Collagenase A (Roche, Basel, Switzerland, Basel, Switzerland) supplemented with 10 U/ml of DNase I for 40 min at 37°C prior to Ficoll-Hypaque gradient centrifugation. Isolation of tissue/tumor-infiltrating leukocytes was done according to the method described earlier (19).

### Flow Cytometry

The following fluorescent dye-conjugated anti-human antibodies were used for staining: anti-VISTA (730804) (R&D Systems, Minneapolis, MN, USA); anti-CD45 (HI30), Streptavidin-BV421, anti-CD3 (OKT3), anti-CD56 (HCD56), anti-Perforin (dG9), anti-TNFα, and anti-IFN-γ (BioLegend, San Diego, CA, USA); anti-PD-L1 (MIH1) and anti-CD8 (SK1) (BD Biosciences, San Jose, CA, USA); and anti-Granzyme B (GB11) (Invitrogen, Carlsbad, CA, USA). For intracellular cytokine staining, cells were stimulated with phorbol-12-myristate-13-acetate (PMA) (50 ng/ml, Sigma-Aldrich, St. Louis, MO, USA) and ionomycin (500 ng/ml, Sigma-Aldrich, MO, USA) in the presence of Brefeldin A (GolgiPlug, BD Biosciences) for 4 h prior to staining with antibodies against surface proteins followed by fixation, permeabilization, and staining with antibodies against intracellular antigens. Cells were acquired on an LSRFortessa (BD) flow cytometer, and data were analyzed using FlowJo X. Dead cells were excluded based on viability dye staining (fixable viability dye eF506, eBioscience, San Diego, CA, USA).

### Generation of Anti-Human VISTA Blocking Antibody

VISTA KO mice (6- to 8-week-old) were immunized by subcutaneous injection in the hind foot pads with human VISTA-mIgG2a-Fc fusion protein. For one animal, 10 μg of VISTA-mIgG2a-Fc fusion protein in 50 μl of phosphate-buffered saline (PBS) was mixed with 50 μl of complete Freund adjuvant (CFA; Sigma-Aldrich, Cat# F6881). Mice were immunized 5 times every 3 days. At 3 days after the final boost, the draining lymph nodes were carefully dissected out. The lymphocytes were fused with Ag8.653 myeloma cells (Sigma-Aldrich, Cat# 85011420) with PEG1500 (Polyethylene Glycol 1500, Roche TM, Cat# 783641, 10 × 4 ml in 75 mM of HEPES, PEG 50% w/v) and cloned with HAT selection (Sigma Cat# H0262) and Hybridoma Fusion and Cloning Supplement (HFCS; 50×, Roche Cat# 11-363-735-001). Hybridoma supernatants were screened for the production of antibodies that can bind to human VISTA-hIgG1-Fc by ELISA and flow cytometry on 293T cells transfected with human VISTA. The positive wells were subcloned by limiting dilution. 311-H7 was selected based on the binding assays, and its isotype was determined as mouse IgG1.

### *Ex Vivo* Culture of Tumor Slices

Freshly resected human kidney tumors were cored and minced into 1-mm^3^ slices, and tumor slices were suspended with RPMI-1640 medium plus 5% human AB serum from healthy male AB donors in the United States (GemCell; Gemini Bio Products, West Sacramento, CA, USA). Tumor slices were placed in 24-well culture plates at 1 ml per well. Anti-VISTA (Clone 311H7, X-KANG United Biopharmaceutical Science & Technology Company, Beijing, China), anti-PD-1 (Clone EH12.2H7, BioLegend), or control antibody (Clone MOPC-21, Bio X Cell, Lebanon, NH, USA) was added into the indicated wells at the concentration of 10 μg/ml for 3 days. Phytohemagglutinin (PHA) measuring 5 μg/ml was included as a positive control. The plates were cultured in a humidified incubator at 37°C with 5% CO_2_. After culturing, supernatants were collected on day 2 and day 3 for cytokine detection, while the tissue was harvested and processed for fluorescence-activated cell sorting (FACS) staining on day 3.

### Enzyme-Linked Immunosorbent Assay

The levels of TNF-α (catalog no. 88-7346-88) and IFN-γ (catalog no. 88-7316-88) were examined using the Ready-SET-Go ELISA assay kit from eBioscience according to the provided description and modification. Briefly, 96-well microtiter plates were coated with the capture antibody. After overnight incubation, plates were washed and blocked for 1 h. After extensive washing, the diluted supernatants collected from *ex vivo* culture and standard solutions were added to the appropriate wells and incubated for 2 h. Detection antibodies were then added. After incubation with Avidin–horseradish peroxidase (HRP), the plates were read at 450 nm by an ELISA reader.

### Statistical Analysis

Statistical analysis was performed with GraphPad Prism 8.0. Statistical comparisons were determined with one-way ANOVA analysis followed by multiple comparisons or non-parametric Mann–Whitney test. Student’s t-test was performed for two-group analysis. *p* < 0.05 was considered to be statistically significant.

## Results

### Generation of One Neutralizing Antibody Against Human VISTA

**Figure 1:**
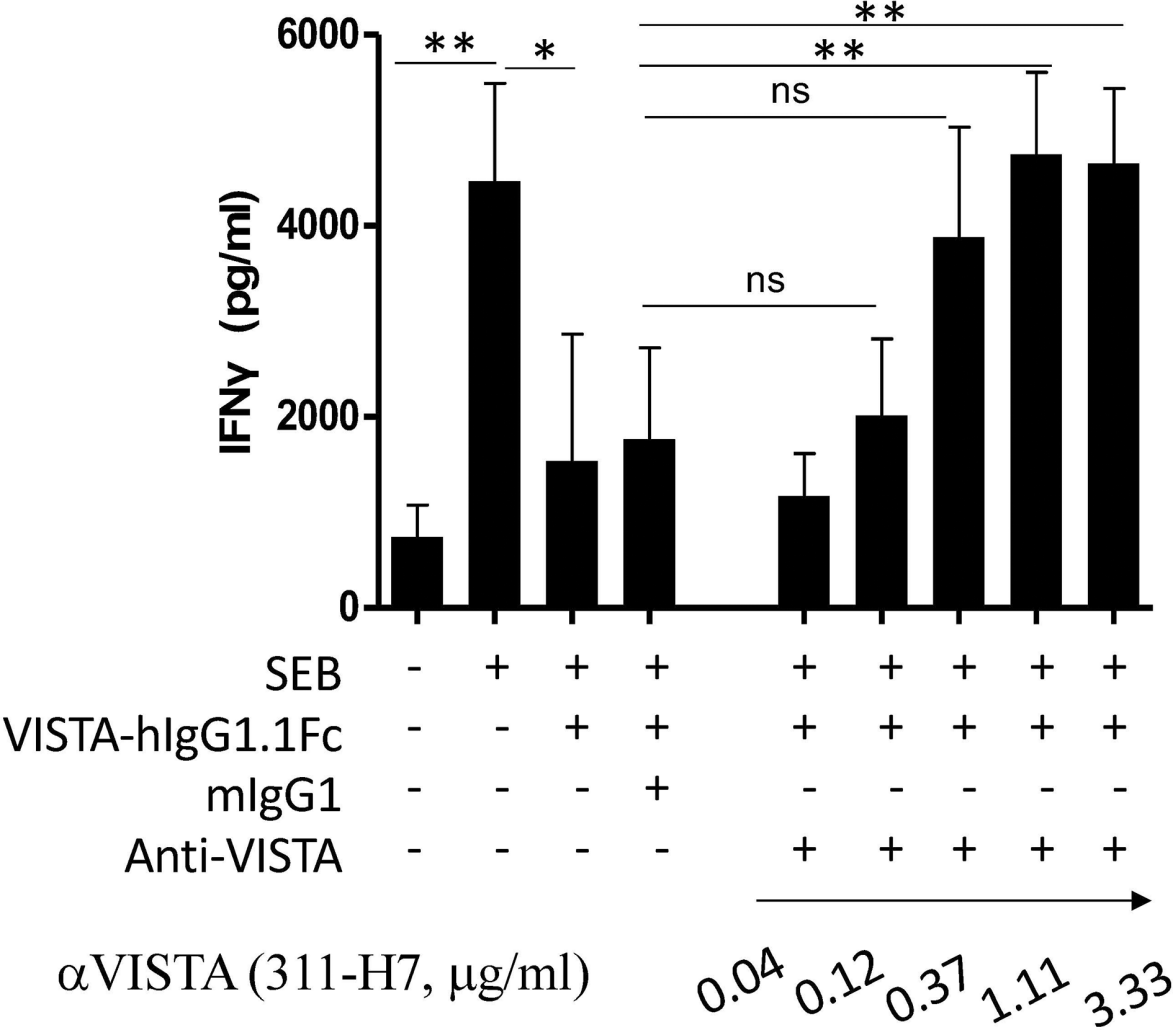
Generation of one neutralizing monoclonal antibody against human VISTA. Peripheral blood mononuclear cells (PBMCs) were cultured in the presence or absence of superantigen staphylococcal enterotoxin B (SEB), and the culture supernatant was analyzed by ELISA for IFN-γ secretion. VISTA-hIgG1.1Fc, mIgG1, and 311-H7 were added into the indicated wells. The experiment was done in triplicate. Four independent experiments show similar results. *p < 0.05, **p < 0.01. ns, not significant.

To explore the contribution of the VISTA signaling pathway to ccRCC progression, one neutralizing antibody against human VISTA was generated *in-house*. As shown in **Figure 1**, the superantigen staphylococcal enterotoxin B (SEB) activated T cells to produce a high level of IFN-γ in a peripheral blood mononuclear cells (PBMC) culture system, while VISTA-hIgG1.1Fc suppressed SEB-mediated T-cell activation, which was consistent with previous reports that VISTA signal inhibits T-cell activation (11). As expected, 311-H7 could restore VISTA-hIgG1.1Fc-mediated IFN-γ reduction at the concentration of 0.12 μg/ml, and this effect was dose-dependent, suggesting that 311-H7 is a neutralizing antibody against human VISTA.

### VISTA and PD-1 Expressions in Treatment-Naïve Patients With Clear Cell Renal Cell Carcinoma

**Figure 2:**
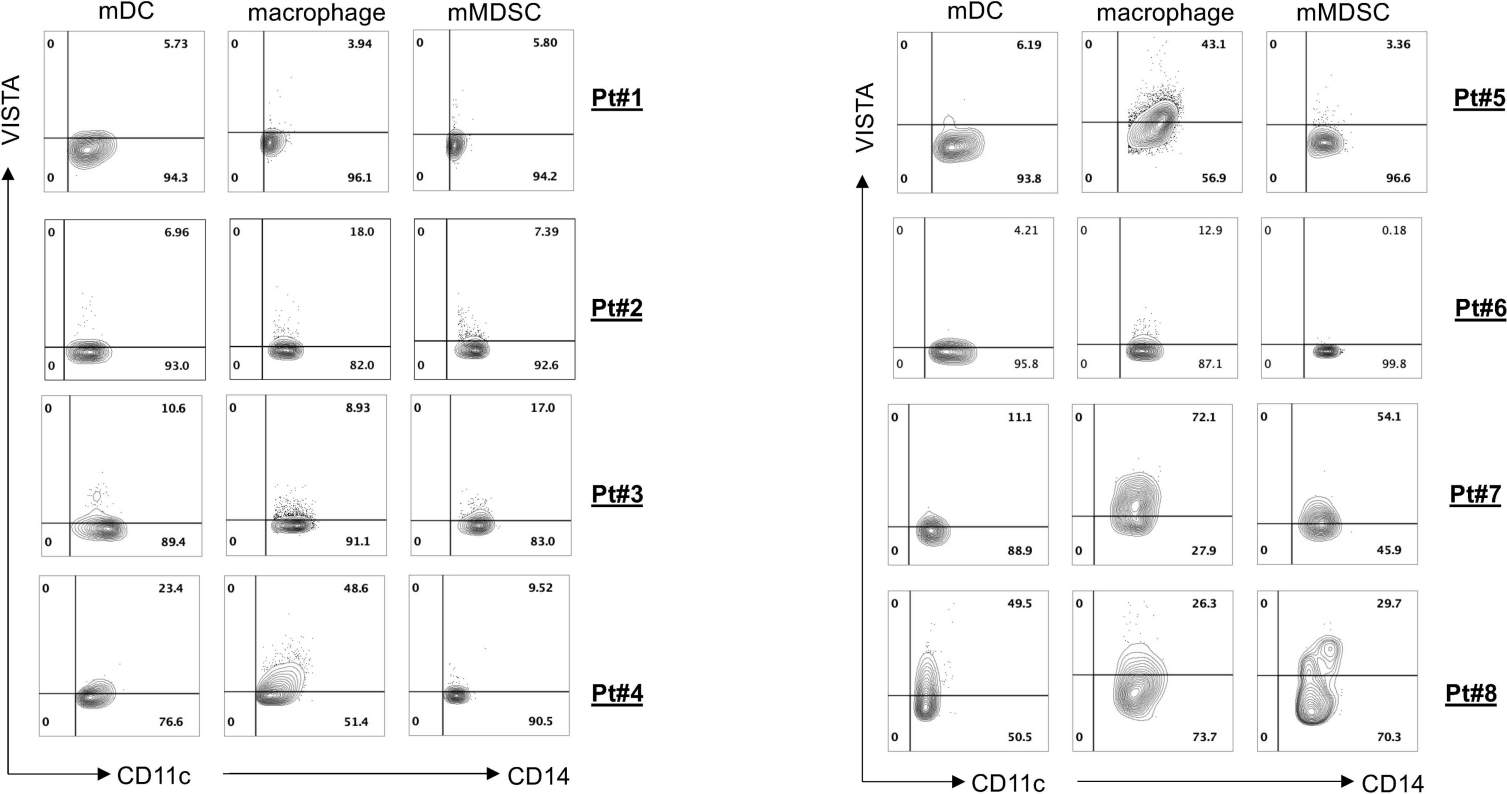
VISTA expression on various myeloid cell subsets. Tumor-infiltrating leukocytes were isolated from fresh tumors of 8 clear cell renal cell carcinoma (ccRCC) patients and then stained with different antibodies against the cell surface. Fluorescence-activated cell sorting (FACS) plot showed VISTA expression on different myeloid cell subsets.

**Figure 3:**
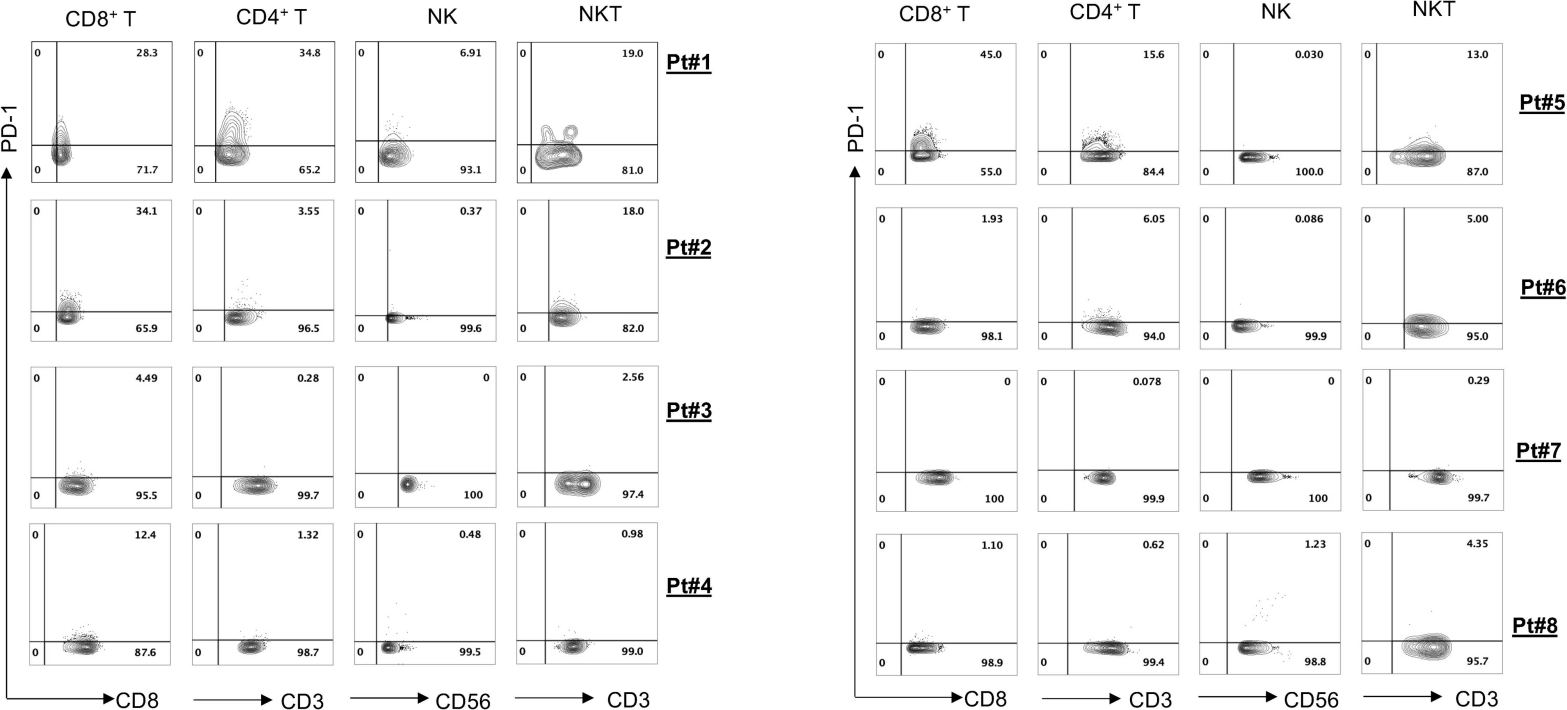
PD-1 expression on various lymphocyte cell subsets. Tumor-infiltrating leukocytes were isolated from fresh tumors of 8 clear cell renal cell carcinoma (ccRCC) patients and then stained with different antibodies against the cell surface. Fluorescence-activated cell sorting (FACS) plot showed PD-1 expression on different lymphocyte subsets.

To evaluate antitumor efficacies of 311-H7, we assessed 8 treatment-naïve patients with ccRCC. The clinical and pathological characteristics of these ccRCC patients are shown in **Table 1**. Of them, 62.5% (5/8) are male. The expressions of PD-1 and VISTA in these patients were measured by the flow cytometry approach. The gating strategy is shown in **Figure S1**. Pt#7 showed the highest VISTA expression levels on macrophages as well as monocytic myeloid-derived suppressor cells (mMDSCs), while Pt#8 displayed the highest VISTA expression level on myeloid dendritic cells (**Figure 2**). We also observed obvious PD-1 expression on lymphocytes in four patients (Pt#1, 2, 4, and 5). Pt#6 expressed weak PD-1 on CD4^+^ T cells and NKT cells (**Figure 3**).

**Table 1:** Clinical characteristics of the ccRCC patients.

Patient no.	Gender	Age	TNM stage	Tumor
				stage
Pt#1	Female	58	T2N0M1	III
Pt#2	Male	85	T2N1M0	II
Pt#3	Male	35	T1N0M0	II
Pt#4	Female	53	T2N0M1	II
Pt#5	Male	67	T2N0M1	II
Pt#6	Female	68	T1N0M0	II
Pt#7	Male	46	T1N0M0	II
Pt#8	Male	61	T2N0M1	II

ccRCC, clear cell renal cell carcinoma.

### Establishment of an *Ex Vivo* Culture Model

**Figure 4:**
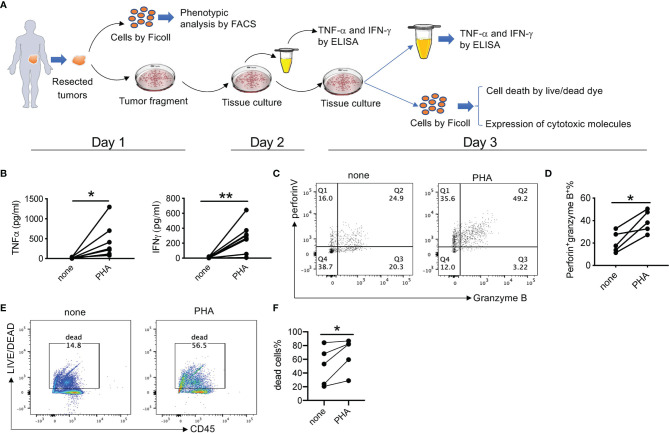
*Ex vivo* culture of clear cell renal cell carcinoma (ccRCC) tissue slices. **(A)** Schematic overview of the *ex vivo* culture model. Human tumor tissue was manually processed in tissue slices with a proportion for single-cell preparation. The tumor tissue slices were placed in a 24-well plate in the presence or absence of different stimuli or inhibitors for 3 days. The culture supernatant was collected on both day 2 and day 3 for cytokine secretion. On day 3, single-cell suspension was prepared and analyzed for cell death, proliferation, and cytotoxic capacity. **(B)** Summarized data about TNF-α and IFN-γ stimulated by phytohemagglutinin (PHA) from 8 patients. The experiment was performed in triplicate. Each line means one patient. **(C)** Representative fluorescence-activated cell sorting (FACS) plot showing expression levels of perforin and granzyme B **(D)** Summarized data about the frequency of perforin^+^ granzyme B^+^ CD8^+^ T cells (n = 5). **(E)** Representative FACS plot showing percentages of dead cells. **(F)** Summarized data about the percentages of dead cells (n = 5). *p < 0.05, **p < 0.01.

We then established an *ex vivo* culture system to evaluate the efficacy of 311-H7 in ccRCC immunotherapy, a means of testing under conditions closer to the physiological reality than isolated cells (**Figure 4A**). PHA was used to identify whether the model was working. Culture supernatant was collected for measurement of TNF-α and IFN-γ on day 2. As shown in **Figure 4B**, PHA treatment induced the tumor slices from all 8 patients to produce significant levels of TNF-α and IFN-γ compared with no treatment. In addition, after 3-day culture, we isolated the mononuclear cells from those tumor slices and stained them with antibodies against cytotoxic molecules. As expected, PHA treatment also induced CD8^+^ T cells to co-express higher levels of perforin and granzyme B than no treatment (**Figures 4C, D**). Notably, this *in vitro* processing of tumor specimens, such as mincing tumor, 3-day culture of tumor slice, and isolation of tumor-infiltrating lymphocytes, led to cell death, which was observed in non-treatment control. However, compared to the control groups, PHA treatment further promoted cell death (**Figures 4E, F**). Taken together, these findings indicated that the *ex vivo* tumor slice culture system works and can be used to test the antitumor efficacy of anti-VISTA antibodies.

### Antitumor Efficacy of the VISTA Inhibitor for Clear Cell Renal Cell Carcinoma Immunotherapy

**Figure 5:**
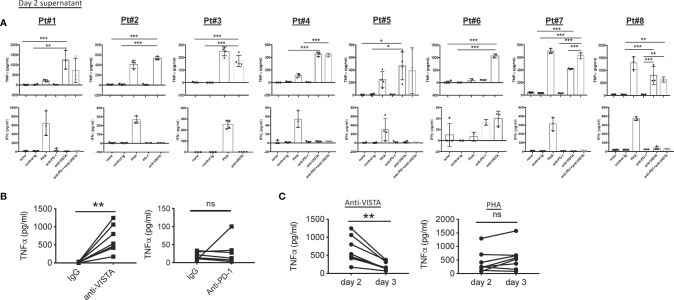
Blockade of VISTA enhanced TNF-α secretion in all tested clear cell renal cell carcinoma (ccRCC) patients. **(A)** Fresh tumor slices from ccRCC patients were stimulated with blocking or control antibodies. Culture supernatants were collected after 48 h Levels of TNF-α and IFN-γ were analyzed by ELISA. TNF-α and IFN-γ secretion on day 2 is shown. **(B)** TNF-α secretion induced by anti-VISTA or anti-PD-1 on day 2. **(C)** Kinetics of TNF-α responses induced by anti-VISTA or PHA. *p < 0.05, **p < 0.01, ***p < 0.001. ns, not significant.

**Figure 6:**
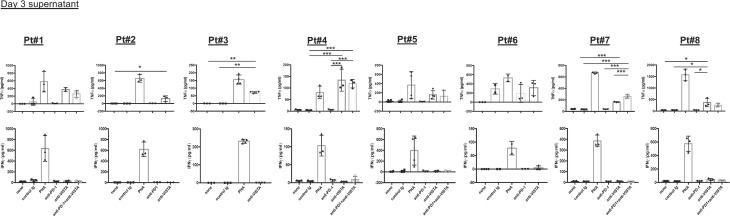
Blockade of VISTA enhanced TNF-α secretion in all tested clear cell renal cell carcinoma (ccRCC) patients on day 3. Fresh tumor slices from ccRCC patients were cultured with blocking or control antibodies. Culture supernatants were collected after 72 h. Levels of TNF-α and IFN-γ were analyzed by ELISA. TNF-α and IFN-γ secretion on day 3 is shown. *p < 0.05, **p < 0.01, ***p < 0.001.

To evaluate the antitumor effectiveness of 311-H7, the tumor slices were cultured in the presence of 311-H7, anti-PD-1, 311-H7 plus anti-PD-1, or isotype control antibody for 2–3 days (**Figures 5** and **6**). The groups in each experiment were set up based on tumor size. 311-H7 treatment also led to significantly increased TNF-α secretion by all the tested patients compared to control isotype antibody, while only Pt#6 produced IFN-γ in response to 311-H7 (**Figures 5A, B**). However, compared with the control antibody, 1 out of 7 tumors (Pt#6) responded weakly to the anti-PD-1 antibody. Notably, TNF-α induced by 311-H7 treatment on day 2 was higher than that on day 3 (**Figure 5C**), but PHA treatment resulted in a similar level of TNF-α production on both days, implying that anti-VISTA might target TAMs and induce their apoptosis. We also observed a synergic effect on TNF-α production by combination of VISTA and PD-1 blockades in 1 out of 5 patients (Pt#7) who had the highest expression level of VISTA on macrophages and mMDSCs on day 2 and day 3 (**Figures 5A**, **6**). In addition, the anti-VISTA treatment also resulted in an enhanced percentage of granzyme B^+^ Perforin^+^ CD8^+^ T cells compared to isotype control in the majority of ccRCC patients (data not shown). Thus, these findings indicated that VISTA could be a promising target for ccRCC immunotherapy.

## Discussion

This study demonstrates that anti-VISTA treatment elicits a TNF-α response in all tested ccRCC patients and a weak IFN-γ response in a small fraction of patients. Of note, co-blockade of VISTA and PD-1 also induced a synergetic effect in 20% of the patients.

There are many preclinical models for oncological research, such as *in vitro* cell culture and patient-derived xenografting (20, 21). Each model has its specific limitations and advantages. In this study, we used an *ex vivo* tumor culture model to assess the efficacies of anti-VISTA and anti-PD-1 antibodies, which takes only 3 days to finish the evaluation. This tissue slice culture system preserved original tumor morphology, which may provide a useful alternative to the challenging xenografting of human tissue in immunocompromised mice (22–25). Arjanneke and colleagues (26) developed another *ex vivo* tissue culture model for prostate and bladder cancer. They cultured tumor slices resuspended with 10% FCS in an oxygenated and sealed container system containing 95% O_2_, while we resuspended the tumor slices with 5% human AB serum and cultured them in a humidified incubator at 37°C with 5% CO_2_ (21% O_2_), which is much closer to the physiological reality.

To date, there are three clinical trials targeting VISTA (NCT02671955, NCT02812875, and NCT04475523). NCT02671955 is a first-in-human phase 1 trial of JNJ-61610588, a fully human IgG1 Kappa anti-VISTA monoclonal antibody (mAb) in subjects with advanced cancer. NCT04475523 is a phase I study of CI-8993 anti-VISTA mAb. NCT02812875 is a study of CA-170 (a small molecule) directly targeting the PD-L1/PD-L2 as well as VISTA immune checkpoints in adult patients with advanced solid tumors or lymphomas who have progressed or are non-responsive to available therapies and for which no standard therapy exists. One recent report showed no direct binding between CA-170 and PD-L1 (27), implying that CA-170 functions through blockade of the VISTA signaling pathway. In the murine lung cancer model, CA-170 showed potent antitumor efficacy (28).

Before VISTA inhibitors will be used as a drug for cancer patients, the VISTA signaling pathway should be deciphered. Notably, VISTA can function as a ligand expressed on myeloid cells and a receptor expressed on CD4^+^ T cells, especially FOXP3^+^ CD4^+^ T cells (29, 30). Recently, Wang et al. found that VSIG-3 acts as a potential ligand of VISTA (31), which requires further validation. Of interest, blockade of VISTA also enhanced TNF-α secretion by ccRCC tumors, but IFN-γ secretion was less affected. However, Lines et al. reported that VISTA-Ig significantly decreased secretion of TNF-α and IFN-γ by CD8^+^ T cells (11). This could be due to different target cells. VISTA-Ig is bound to its receptors, which are expressed on CD8^+^ T cells and in turn reduced cytokine production in CD8^+^ T cells, such as IFN-γ. In our study, intratumoral VISTA was highly expressed on TAMs. Thus, anti-VISTA mainly targeted TAMs and induced them to produce TNF-α.

TNF-α is a pro-inflammatory cytokine that was mainly produced by myeloid-derived cells such as monocytes, macrophages, and dendritic cells. Many other cells such as T cells and endothelial cells can also produce it under stress conditions. Normally, TNF-α secretion by monocytes/macrophages reached a peak at an early time point (approximately 8 h) and then gradually decreased, which was because TNF-α could induce apoptosis of monocytes/macrophages, which resulted in the degradation of TNF-α (32–34). This could be why the TNF-α level by anti-VISTA on day 2 supernatant was higher than that on day 3.

In a word, VISTA might be a promising target for ccRCC immunotherapy. The cultured tumor slice model, which remains tumor architecture, represents a viable tool to evaluate antitumor efficacy for the inhibitors of immune checkpoints.

### Limitations

The caveats of this study include the sample size and the focus on one neutralizing anti-VISTA antibody. The sample size was limited by expediency, and our conclusion, thus required further confirmation in a large cohort of ccRCC patients. In addition, the tumor specimens in this study were treatment-free, and it would be also interesting to assess antitumor immune responses for tumors from patients who had already been treated in this *ex vivo* culture model.

Although we detected PD-1 and VISTA expressions before tissue culture, we did not analyze their expressions during and after culture. If one new technology could allow us to detect real-time PD-1 and VISTA expressions during culture, it would be very informative. Here cell viability was investigated. If permitted, a cell proliferation marker, such as Ki-67, should be incorporated to elucidate cell proliferation after a 3-day *ex vivo* culture. If the tumor is large enough, the tumor structure before and after *ex vivo* tissue culture should be assessed to elucidate whether the *ex vivo* culture leads to the maintenance of tissue architecture. Moreover, immunofluorescence staining with antibodies against signature markers of immune cells allows us to explore whether the *ex vivo* culture could change their locations.

Lastly, 311-H7 (anti-human VISTA) was used to evaluate this *ex vivo* culture model. Other VISTA inhibitors, especially the ones in clinical trials, if possible, would be used in order to validate this model. Of note, this *ex vivo* culture tool cannot predict or reflect clinical response *in vivo* in ccRCC patients treated by immune checkpoint inhibitors.

## Data Availability Statement

The original contributions presented in the study are included in the article/**Supplementary Material**. Further inquiries can be directed to the corresponding authors.

## Ethics Statement

The studies involving human participants were reviewed and approved by the institutional review board at Tsinghua University. The patients/participants provided their written informed consent to participate in this study.

## Author Contributions

SH performed most of the experiments. TS, YD, and LG produced an anti-human VISTA neutralizing antibody. TX performed some of the experiments. HX, WH, and ZZ collected clinical specimens. QY and JL supervised clinical specimens. LN wrote the manuscript. LN and CD designed and supervised the study. All authors listed have made a substantial, direct, and intellectual contribution to the work and approved it for publication.

## Funding

This work was supported by grants from the National Natural Science Foundation of China (NSFC) (31991173 and 31991170), Tsinghua University Spring Breeze Fund (2020Z99CFG008), NSFC general project (81570679), Beijing NOVA program (Z161100004916141), Beijing Natural Science Foundation (L212051 and Z200027), and Tsinghua University-Xiamen Chang Gung Hospital Joint Research Center for Anaphylactic Disease.

## Conflict of Interest

YD, TS, and LG are employees of Kanova Biopharmaceutical Co., Ltd.

The remaining authors declare that the research was conducted in the absence of any commercial or financial relationships that could be construed as a potential conflict of interest.

## Publisher’s Note

All claims expressed in this article are solely those of the authors and do not necessarily represent those of their affiliated organizations, or those of the publisher, the editors and the reviewers. Any product that may be evaluated in this article, or claim that may be made by its manufacturer, is not guaranteed or endorsed by the publisher.

## Abbreviations

RCC, renal cell carcinoma; ccRCC, clear cell renal cell carcinoma; FDA, Food and Drug Administration; TAMs, tumor-associated macrophages; SEB, superantigen staphylococcal enterotoxin B; PBMC, peripheral blood mononuclear cell; mMDSCs, monocytic myeloid-derived suppressor cells; PHA, phytohemagglutinin; PMA, phorbol-12-myristate-13-acetate.
