# Supplementary figures and images for: Establishment of an Ex Vivo Tissue Culture Model for Evaluation of Antitumor Efficacy in Clear Cell Renal Cell Carcinoma
Source: Front Oncol. 2022 Apr 6;12:851191. doi: 10.3389/fonc.2022.851191 (PMC9019348; doi:10.3389/fonc.2022.851191)

## Supplementary Figure legends

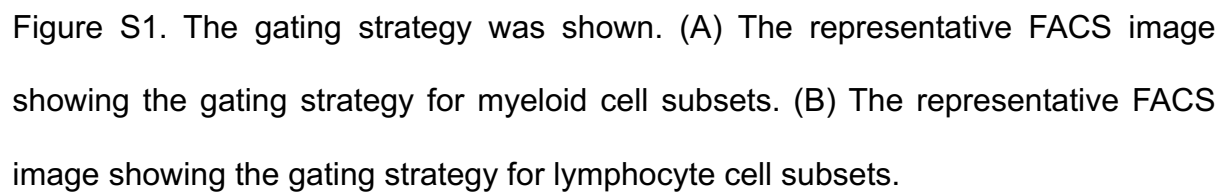

Supplement: Supplementary file 1 [file DataSheet_1.pdf]
